# VMXm – A sub-micron focus macromolecular crystallography beamline at Diamond Light Source

**DOI:** 10.1107/S1600577524009160

**Published:** 2024-10-30

**Authors:** Anna J. Warren, Jose Trincao, Adam D. Crawshaw, Emma V. Beale, Graham Duller, Andrew Stallwood, Mark Lunnon, Richard Littlewood, Adam Prescott, Andrew Foster, Neil Smith, Guenther Rehm, Sandira Gayadeen, Christopher Bloomer, Lucia Alianelli, David Laundy, John Sutter, Leo Cahill, Gwyndaf Evans

**Affiliations:** a Diamond Light Source Harwell Science and Innovation Campus Didcot OxfordshireOX11 0DE United Kingdom; b Paul Scherrer Institut Forschungsstrasse 111 5232 Villigen PSI Switzerland; c Rosalind Franklin Institute Rutherford Appleton Laboratory Harwell Campus Didcot OxfordshireOX11 0QX United Kingdom; d Helmholtz-Zentrum Berlin Hahn-Meitner-Platz 1 14109 Berlin Germany; University of Essex, United Kingdom

**Keywords:** microfocus, in vacuum, SEM, scanning electron microscopy, macromolecular crystallography, beamlines, synchrotron radiation

## Abstract

VMXm joins the suite of operational macromolecular crystallography beamlines at Diamond Light Source. It has been designed to optimize rotation data collections from protein crystals less than 10 µm and down to below 1 µm in size. The beamline has a fully focused beam of 0.3 × 2.3 µm (vertical × horizontal) with a tuneable energy range (6–28 keV) and high flux (1.6 × 10^12^ photons s^−1^ at 12.5 keV). The crystals are housed within a vacuum chamber to minimize background scatter from air. Crystals are plunge-cooled on cryo-electron microscopy grids, allowing much of the liquid surrounding the crystals to be removed. These factors improve the signal-to-noise during data collection and the lifetime of the microcrystals can be prolonged by exploiting photoelectron escape. A novel *in vacuo* sample environment has been designed which also houses a scanning electron microscope to aid with sample visualization. This combination of features at VMXm allows measurements at the physical limits of X-ray crystallography on biomacromolecules to be explored and exploited.

## Abstract 2

The VMXm beamline at Diamond Light Source enables macromolecular micro-crystallography near the physical limits achievable using synchrotron radiation.

## 1. Introduction

Since the introduction of macromolecular micro-crystallography (microMX) in the late 1990s (Perrakis *et al.*, 1999 ▸) and its implementation at several synchrotron beamline instruments worldwide (Smith *et al.*, 2012 ▸; Evans *et al.*, 2011*b* ▸; Aragão *et al.*, 2018 ▸; Duran *et al.*, 2013 ▸; Gu *et al.*, 2021 ▸; Hirata *et al.*, 2013 ▸; Nanao *et al.*, 2022 ▸; Schneider *et al.*, 2021 ▸; von Stetten *et al.*, 2020 ▸; Chavas *et al.*, 2024 ▸), microMX has become a staple of macromolecular crystallographers for determining atomic structures in cases where crystals are very small or weakly diffracting (Ji *et al.*, 2010 ▸; Hollenstein *et al.*, 2013 ▸) or highly inhomogeneous (Peselis & Serganov, 2018 ▸; Bowler *et al.*, 2010 ▸). X-ray free-electron lasers (XFELs) have also been used to study small and weakly diffracting crystals, but their extremely high peak brilliance obliterates the crystals, or for larger crystals destroys the volume of sample intersected by the beam; both approaches are known as ‘diffraction before destruction’ (Chapman *et al.*, 2011 ▸). In combination with this, liquid jet based sample delivery methods are often employed, where the delivery rate of crystals to the beam is matched to the repetition rate of the XFEL pulse rate; however, this can be hard to achieve (Weierstall, 2014 ▸; Chapman *et al.*, 2011 ▸). XFEL experiments therefore typically require larger quantities of sample than synchrotron measurements and employ serial crystallography approaches.

Large amounts of sample can sometimes be difficult, expensive and time consuming to produce. For the well characterized samples often used at XFELs to study protein dynamics (Nango *et al.*, 2016 ▸; Neutze *et al.*, 2015 ▸) or electron transfer mechanisms (Kern *et al.*, 2018 ▸; Young *et al.*, 2016 ▸), the design of protocols for the production of large volumes of crystals in solution was a significant and critical aspect of the experiment. For less well characterized systems, the challenge of sample production makes X-ray structure determination approaches that are efficient in sample usage and rich in information an attractive prospect for structural biologists. At the other end of the microMX scale, microcrystal electron diffraction (microED) is developing rapidly into a method to collect diffraction data from nanoscale crystals (Acehan *et al.*, 2024 ▸). However, due to the inelastic mean free path of the electron beams at the energies currently available from commercial transmission electron microscopes (100–300 kV), the sample thickness envelope has an upper limit of ∼300 nm (Martynowycz *et al.*, 2021 ▸; Clabbers & Abrahams, 2018 ▸). As such, many of the protein structures determined using microED have needed their crystal thickness reducing with focused ion beam (FIB) milling, adding time-consuming steps to the sample preparation pipeline as well as needing access to FIB–SEM (scanning electron microscopy) instrumentation (Duyvesteyn *et al.*, 2018 ▸; Li *et al.*, 2018 ▸; Martynowycz & Gonen, 2021 ▸). The Versatile Macromolecular Crystallography microfocus (VMXm) beamline at Diamond Light Source has been designed and constructed with these considerations in mind.

The application of synchrotron microbeams to macromolecular crystallography (MX) (Evans *et al.*, 2011*b* ▸; Perrakis *et al.*, 1999 ▸), either using microapertures (Fischetti *et al.*, 2009 ▸) or by microfocusing (Smith *et al.*, 2012 ▸), has typically built upon traditional sample environment technologies employing setups in air with cryocooling performed by flowing gaseous nitro­gen at 100 K over samples typically mounted in Kapton loops, micromeshes (MiTeGen, USA) or litholoops (Molecular Dimensions, UK). These setups have intrinsically imposed limits on the minimum crystal size and diffracting resolution for any given system using microMX. These limitations are mainly due to radiation damage of the microcrystals and background X-ray scattering generated by beamline apparatus, the air path and the sample. Other practical limitations of studying crystals less than 5 µm in size include sample visualization and alignment, beam and sample stability, and the handling and mounting of such small crystals.

As X-ray diffraction intensities become weak with micron sized crystals, X-ray background becomes the dominating influence on signal-to-noise (Holton & Frankel, 2010 ▸). The benefits of improving the overall signal-to-noise ratio in microcrystal diffraction data include increasing the observable resolution, and/or increasing the lifetime of the microcrystals in the X-ray beam by reducing the dose on the crystals to still obtain comparable diffraction. Increasing data resolution could bring benefits in terms of biological interpretability of electron-density maps, reducing potential ambiguity of results. Increasing microcrystal lifetime would increase the data quantity from a single crystal and reduce the total number of crystals required for a complete data set. For multiple crystal or serial crystallography data sets, completeness, multiplicity, number of crystals, resolution and data quality can all be traded against each other. By expanding what is measurable from any single crystal, improvements in some or all metrics are possible.

A reduction in background scattering can be achieved by matching the beamsize to the crystal size (Evans *et al.*, 2011*a* ▸; Nave, 2014 ▸). This removes much of the scattering from the material surrounding the crystal, thus increasing the signal-to-noise ratio. Moreover, other contributions to background are the air paths before and after the sample, up to the beamstop, and the nitro­gen cryo-gas stream. Through alignment of the pre-sample apertures, and bringing these and the beamstop into close proximity to the sample, a reduction in scatter has been observed (Madsen *et al.*, 1999 ▸). Background X-ray scatter has also been shown to be reduced by replacing the nitro­gen cryo-gas stream with a helium cold gas stream (Glaeser *et al.*, 2000 ▸). To ultimately reduce the air paths as much as possible, whole sample environments can be placed under vacuum or a helium environment (Perutz & Rogers, 1946 ▸; Krieger & Stroud, 1976 ▸; Liu *et al.*, 2001 ▸; Hirata *et al.*, 2013 ▸; Djinovic Carugo *et al.*, 2005 ▸; Hendrickson & Ogata, 1997 ▸; Wagner *et al.*, 2016 ▸; Basu *et al.*, 2019 ▸). The only remaining contributions to the scattered X-rays come from the sample, sample mount, liquid surrounding the crystals and any scatter from upstream beamline components such as apertures.

Radiation damage can be a limiting factor in standard rotation data collections for microcrystals as a higher dose is required to obtain similar information to larger crystals. However, simulations have shown that, for crystals smaller than 10 µm with minimal surrounding liquid, the photoelectrons produced in the crystal are able to escape, causing less secondary damage within the crystal, thereby increasing its lifetime. This is improved further by collecting at higher energies (up to 40 keV) as the photoelectron path would be greater, with even less energy deposited in the crystal (Cowan & Nave, 2008 ▸; Nave & Hill, 2005 ▸), although it was noted that detector technology would also need to improve to allow good quantum efficiency at these higher energies. This phenomenon was demonstrated on larger crystals with beamsizes ranging from 15.6 to 0.84 µm, at 18.5 keV. It was shown here that radiation damage per calculated dose decreased threefold over this crystal size range as the higher-energy photoelectrons were escaping the area where the beam had been exposing the crystal (Sanishvili *et al.*, 2011 ▸). This was investigated further with Monte Carlo simulations, factoring in photoelectron escape, the use of higher energies and different detector sensors. At higher energies, small crystal sizes (5 µm or less) and similarly small beamsizes, photoelectron escape becomes more probable, thus significantly decreasing the dose per diffracted photon. This effect can be exploited further through the use of detectors with cadmium telluride (CdTe) sensors where the quantum efficiency is consistent across higher energies (optimally around 26 keV) (Dickerson & Garman, 2019 ▸). This has recently been shown experimentally, with smaller microcrystals (5 × 3 × 3 µm) having a 51% increase in their *D*_1/2_ when collecting at 20.1 keV versus 13.5 keV. When this is compared with the larger crystals (20 × 8 × 8 µm) at both energies, little variation in the *D*_1/2_ value is observed (Storm *et al.*, 2020 ▸). These measurements were possible using a PILATUS3 detector (DECTRIS) with a CdTe sensor which displays good quantum efficiency at the compared energies (Zambon *et al.*, 2018 ▸). This was further confirmed with the use of an EIGER2 X CdTe detector (DECTRIS), looking at comparisons with a PILATUS3 X silicon (Si) detector. Improvements were observed in the mean diffracted intensity and also in resolution as the energy was increased from ∼12 keV to ∼25 keV (Storm *et al.*, 2021 ▸).

The VMXm beamline at Diamond Light Source has been designed, built and commissioned to address the issues described above and to optimize rotation data collection from crystals smaller than 10 µm in size. This article outlines VMXm and the equipment used to help facilitate a fully focused beam of 0.3 × 2.3 µm (vertical × horizontal) at the sample position, which can be further reduced in the horizontal direction to 1.3 µm using slits. The flux of the focused beam at the sample position reaches 1.6 × 10^12^ photons s^−1^ at 12.5 keV. The crystals are mounted on cryo-electron microscopy (cryoEM) grids, minimizing the volume of crystal suspension required while also minimizing the surrounding mother liquor. The samples are housed in a novel *in vacuo* sample environment and visualized using an on-axis optical microscope, with a scanning electron microscope integrated to aid with sample alignment in the future. The beamline is equipped with an EIGER2 X 9M CdTe detector (DECTRIS) to exploit photoelectron escape from the microcrystals, as well as a PILATUS3 6M Si detector (DECTRIS) for conventional energies. The combination of an optimized beam at the sample position, the sample preparation and sample environment results in a larger quantity and higher quality of rotation data being measured from each crystal.

## 2. Beamline overview

### 2.1. Diamond storage ring, DDBA parameters and undulator

When VMXm was conceived, no available straight sections remained on the Diamond storage ring, but the high brightness of an insertion device was essential to meet the beamline specifications. A solution was developed to locally modify the magnetic lattice at bending-magnet sector B02 to create a new straight section, a so-called mid-straight. The original B02 double-bend achromat arrangement was adapted into a double-double-bend achromat (DDBA) scheme that permitted the introduction of a 2 m-long U21 in-vacuum undulator between each of the achromat pairs while still using the existing ratchet wall radiation exit pipe. This has been described elsewhere in detail (Bartolini *et al.*, 2018 ▸). The introduction of the DDBA straight offered some limited choice of source parameters (at the same emittance) compared with other standard Diamond storage-ring straight sections. The final electron and X-ray source parameters are shown in Table 1 ▸. The U21 in-vacuum undulator has a minimum gap of 5 mm, optimized to emit the highest brilliance at 12.4 keV.

### 2.2. Optics layout

**Figure 1:**
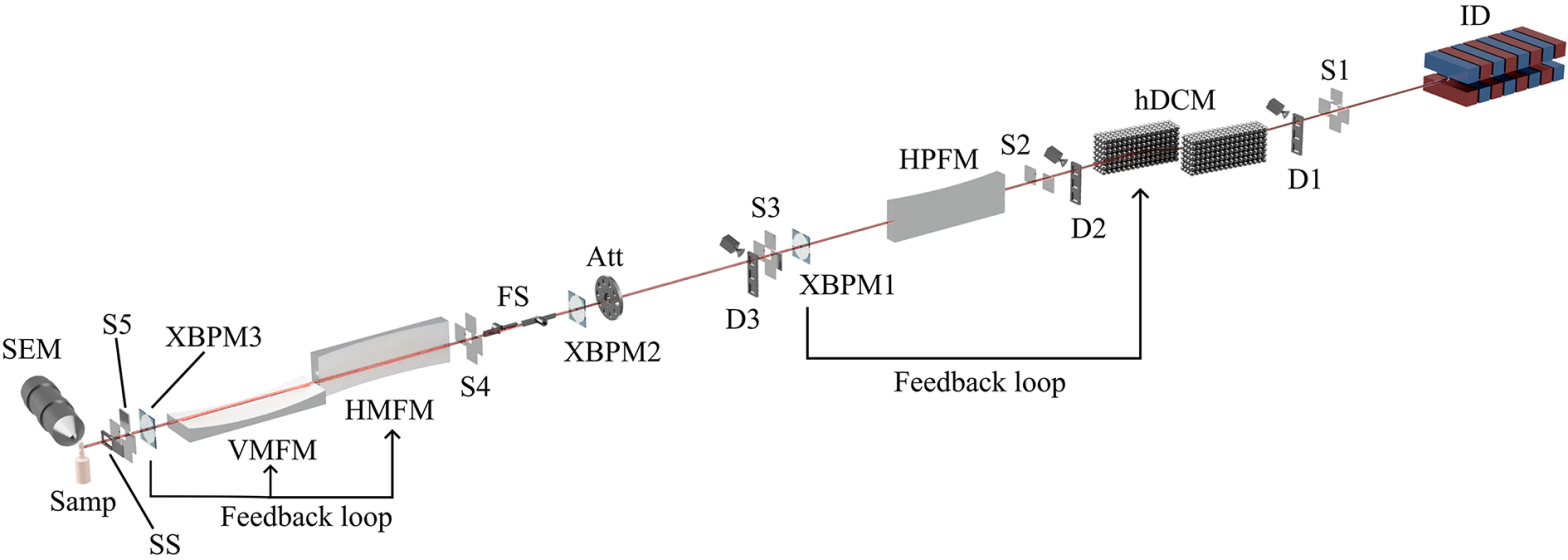
Beamline schematic of VMXm showing the main components. These include the insertion device (ID), beamline slits (S1, S2, S3, S4 and S5), diagnostics (D1, D2 and D3), horizontally deflecting double-crystal monochromator (hDCM), horizontal pre-focusing mirror (HPFM), X-ray beam position monitors (XBPM1, 2 and 3), attenuators (Att), fast shutter (FS), horizontal microfocusing mirror (HMFM), vertical microfocusing mirror (VMFM), slow shutter (SS), sample position (Samp) and scanning electron microscope (SEM). The feedback loops from XBPM1 and 3 to the relevant optics have also been indicated. Several of the components surrounding the sample position have been omitted for clarity. The front-end is positioned between the ID and S1.

The optics for VMXm consist of a horizontal deflecting double-crystal monochromator (hDCM), two horizontal mirrors and a single vertical mirror. In the horizontal there is a two-stage demagnification with a horizontal pre-focusing mirror (HPFM) focusing down to a secondary source, followed by a horizontal microfocus mirror (HMFM). This provides a fully focused beam of 2.3 µm at the sample position, with the option to reduce to 1.3 µm by defining the beam with the secondary source slits. For the vertical, there is a single fixed focal length microfocus mirror (VMFM), with multiple lanes to change beamsize (Laundy *et al.*, 2019 ▸). Here the beam can be changed from 0.3 to 8.7 µm with no reduction to the flux. The beamline can be tuned in the energy range 6–28 keV, with a flux at 12.5 keV at the sample position of the fully focused beam of 1.6 × 10^12^ photons s^−1^, and 1.3 × 10^11^ photons s^−1^ with the reduced horizontal beamsize. The main components of the VMXm beamline are shown in Fig. 1 ▸, with their distance from the source displayed in Table 2 ▸.

#### 2.2.1. Monochromator

The hDCM is positioned 29.85 m from the source, and was chosen for stability reasons, as the small beamsize and distance from the sample position impose strong requirements for positional and rotational stability from the monochromator. The hDCM system was designed and manufactured by Cinel, Italy, and comprises two symmetrically cut Si(111) single crystals. Both crystals are cryocooled, the first crystal directly with liquid nitro­gen flowing to copper heat exchangers and the second indirectly through copper braids. The hDCM operates in the energy range 6–28 keV with a fixed exit (25 mm offset), allowing the energy to be readily changed without realignment of the downstream beamline optics.

Water-cooled primary white beam slits (S1, Bestec, Germany) featuring four individually motorized tungsten blades are placed upstream of the monochromator to define the incident beamsize horizontally and vertically. After the slits is a motorized actuator which can insert a water-cooled CVD (chemical vapour deposition) diamond diagnostic (D1) into the path of the X-ray beam. The fluorescence from this diamond is visualized on a Manta G-235B GigE camera (Allied Vision, Germany). The screen can be inserted and removed as required.

Directly after the hDCM is a diagnostic (D2), for which the actuator can be moved to five different positions. One position is empty to allow the full beam through, the other four positions are occupied with three foils (molybdenum, platinum and iron) for energy calibration of the monochromator, and finally a cerium-doped yttrium aluminium garnet (YAG) scintillator for imaging the beam on a Manta G-235B GigE camera. After this diagnostic, another actuator is positioned which can move a silicon PIN diode (OSI Optoelectronics, USA) into the path of the X-ray beam. This allows intensity and calibration measurements of the hDCM.

#### 2.2.2. Focusing

The specifications and optical parameters for the VMXm mirrors can be seen in Table 3 ▸. Horizontally, focusing is achieved through a two-stage demagnification with a fixed focal length. The first stage is achieved by the HPFM, located 32.5 m from the source, and incorporates a set of horizontal slits (S2) which define the beam prior to the mirror. The HPFM consists of a silicon bimorph mirror (Signorato *et al.*, 1998 ▸) with three lanes (rhodium, bare silicon and platinum), focusing the beam to the secondary source slits (S3) 38.5 m from the source (or 6 m downstream from the centre of the HPFM). The HPFM, S2 and S3 slits were all supplied by Cinel, Italy. A further 10.7 m downstream of the secondary source (at 49.2 m) is the fixed-curvature silicon elliptical HMFM (JTEC, Japan), coated in 5 nm rhodium over 50 nm Pt. This focuses the beam horizontally to the sample position, a further 0.8 m downstream of the centre of this mirror. The beamsize at the sample position is defined by the secondary source S3 slits.

To optimize the secondary source focus as accurately as possible, an X-ray beam position monitor (XBPM1) (38.31 m) is positioned directly before S3 and a diagnostic (D3, 38.71 m) is placed immediately afterwards. The slits are a double piggyback system, allowing for a very precise slit opening, which is maintained during scanning as only one motor is required to move both slit blades simultaneously. XBPM1 is a single-crystal CVD diamond XBPM from Cividec Instrumentation GmbH, Austria. D3 contains an actuator that can position either a silicon diode into the path of the beam, or a cerium-doped YAG scintillator to image the beam with a Manta G-507B GigE camera.

The vertical focus is obtained by a single fixed focal length mirror positioned downstream of the HMFM, 0.4 m from the sample position. The VMFM (JTEC, Japan) is positioned 49.6 m from the source and is an elliptical silicon mirror coated in 5.6 nm rhodium over 55 nm platinum. The mirror has seven discrete lanes, 4 mm in width, each defining a different beamsize at the sample position. Each lane has a different artificially introduced surface profile achieved by the elliptical profile having four added parabolic sections. Each section has a specific amplitude in order to broaden the focus by a defined amount. The vertical beamsize can be changed rapidly by translating the mirror to the required lane (Laundy *et al.*, 2019 ▸). This multi-lane mirror was chosen for vertical focusing to allow rapid interchange between lanes within a few seconds to vary the X-ray beamsize. Mechanically bent mirrors typically take tens of seconds to make large changes to their curvature. Bimorph mirrors with piezo actuators can change the optical curvature within only a few seconds. However, without closed-loop control, the curvature of bimorphs can drift over several minutes caused by piezo creep and reactive strain from the opto-mechanical holder (Alcock *et al.*, 2023 ▸; Alcock *et al.*, 2019*a* ▸; Alcock *et al.*, 2019*b* ▸).

For focusing the beam to the sample position, both the horizontal and vertical beamsize can be adjusted rapidly to meet the requirements of the experiment. In the vertical direction, to change to adjacent lanes on the VMFM takes approximately 6 s, while changing from the smallest to the largest vertical beamsize takes approximately 45 s. Horizontally, to reduce or enlarge the beamsize using the secondary source slits takes approximately 3 s.

### 2.3. Experimental setup

Prior to the microfocus mirrors in the experimental hutch are a pair of slits (S4), a fast shutter, a second Cividec diamond XBPM (XBPM2) and some attenuators. The fast shutter was designed in-house to minimize the rise time of the exposure to X-rays at the sample position. The shutter is composed of two double-bladed rotors actuated by ex-vacuum servo motors capable of accelerating the blades to 10 m s^−1^ through a ferrofluidic seal. Two of these rotors are present, one for opening and one for closing, thus enabling similar rise and fall times (200 µs) and extremely short exposures (∼400 µs). This shutter is positioned prior to the mirrors due to size constraints between the end of the mirror vessel and the sample position. The attenuators are based on an in-house design composed of four wheels, each of the wheels containing ten apertures. One aperture is left empty to allow for unattenuated beam. The remaining nine positions are occupied by varying thicknesses of aluminium or titanium foils.

Directly after the microfocus mirrors is a third Cividec diamond XBPM (XBPM3), a slow shutter and a pair of pre-sample slits (S5). The slow shutter and slits consist of five SmarAct piezo stages (SmarAct GmbH, Germany), each with a single tungsten blade. Individual blades can be moved independently to either define the beam at the sample position or block any beam reaching the sample whilst still illuminating XBPM3 for beam alignment purposes. After the pre-sample slits is a small beryllium window (Materion, USA); this separates the vacuum of the optical beamline components from that of the sample environment. The beryllium window is 100 µm thick with a 6 mm diameter. This is mounted to the back of the sample vacuum vessel where there is a 3 mm opening for the beam to pass through. When vacuum has been lost in the sample vessel, the beryllium window is able to withstand these sharp changes in vacuum pressures.

VMXm is equipped with two detectors, an EIGER2 X 9M with 750 µm CdTe sensor (Donath *et al.*, 2023 ▸) and a PILATUS3 6M with a 1000 µm silicon sensor (DECTRIS, Switzerland). They are both mounted on a detector stage constructed and designed by LG Motion, UK. This allows for movement of the detector in the beam direction from 0.292 m (EIGER) and 0.461 m (PILATUS) up to 4.7 m from the sample position, as well as vertical motion and small horizontal manual adjustments. The vertical motion of the detector stage allows switching of the two detectors as required by the experiment. The EIGER is better optimized for higher-energy data collections (>14 keV) in terms of its quantum efficiency, and also the achievable resolution on the detector. However, at lower energies the resolution limit of this detector is limited, and therefore the PILATUS would be chosen for these.

#### 2.3.1. Sample environment

**Figure 2:**
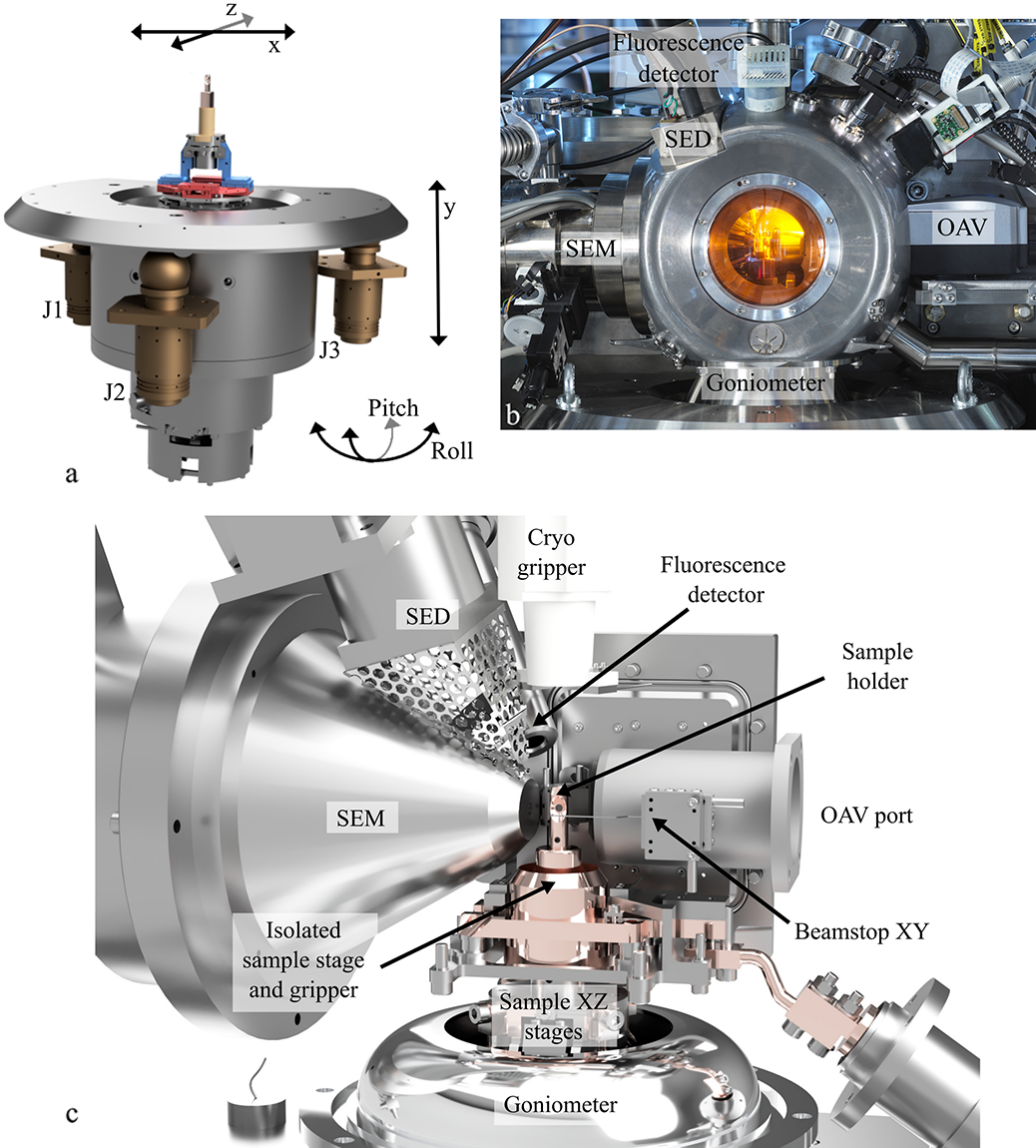
A 3D rendering of the goniometer (*a*), an external image of the VMXm sample vacuum chamber (*b*), equipment within the chamber surrounding the sample environment (*c*). Arrows indicate the direction of travel of the goniometer and sample in (*a*). J1, J2 and J3 denote the three air-bearing slides which are used to align the height of the sample (*y*), as well as the rotation axis in pitch and roll. The red (*z*) and blue (*x*) coloured stages are the SmarAct stages, each with two stages per direction; these are used to align the sample in *x* and *z*. (*b*) and (*c*) show the internal and external configuration around the sample position, including the scanning electron microscope (SEM), secondary electron detector (SED) and on-axis viewing system (OAV).

An image of the VMXm sample vessel, 3D rendering of the sample environment and goniometer are shown in Fig. 2 ▸. The goniometer body is positioned on three air-bearing slides forming a kinematic mount, each with a total vertical travel range of ∼15 mm. There are two sets of vertical motors controlling these, either coarse stepper motors (in-house design) which can achieve larger movements (0–15 mm) with a coarser step size (∼50 nm), or fine piezo stacks (Piezosystem Jena GmbH, Germany) to do smaller fine adjustments (∼5 nm) over a smaller range (0–40 µm). The goniometer itself is an air-bearing goniometer from Fluid Film Devices, UK. The goniometer is capable of continuous rotation with vacuum sealing by our own design of a ferrofluidic seal. This seal and goniometer motor contribute to a very low runout at the sample of around 60 nm (peak to peak, measured at 100° per second over 360°). These measurements were carried out with the goniometer offline in a temperature-controlled environment, with manual stages mounted for alignment of the measuring device.

On top of the goniometer then sits the sample (110 mm from the air-bearing surface), on two sets of stages perpendicular to each other (*x* and *z* movements), each consisting of two SmarAct motion stages. The two stages per direction act together for a stable motion. The sample is held in place on the goniometer by a mechanical collet, designed in-house, which avoids the use of magnets (since they are incompatible with the scanning electron microscope). The sample is thermally isolated from the centring stages. Once the X-ray focal position is known, the rotation axis is aligned and then moved to be coincident to this, through motion of the three vertical slides, either in a coordinated move of pitch or roll (<0.2°). The SmarAct sample stages then allow the centring of the sample to the goniometer rotation axis which is pre-centred to the X-ray focus.

For coarse alignments, or alignment of larger samples, a standard on-axis viewing system (OAV) is used which can resolve crystals as small as 1–2 µm. Directly after the beryllium exit window a drilled mirror with 0.5 mm hole is positioned at a 45° angle to reflect the image of the sample through the window of the OAV port, while allowing the X-ray beam to pass through to the sample. Behind the window, in air, a 10× objective lens, 12× Fetura zoom lens unit and 1× lens tube (Qioptiq Photonics GmbH, Germany) are positioned, coupled to a Manta G-895C camera. These are mounted on a stage which can be manually adjusted for alignment. For higher-resolution sample visualization (smaller than 1 µm), a scanning electron microscope (JSM-IT100, Jeol, Japan) column is installed in the horizontal plane 80° from the primary beam axis. The sample environment must be operated under vacuum (better than 10^−4^ mbar, usually operated in the range 10^−5^–10^−6^ mbar) to enable the use of the scanning electron microscope. This enforced vacuum environment has the additional benefits of minimizing air scatter for diffraction experiments and eliminating convection. Positioned directly above the scanning electron microscope column is the secondary electron detector (SED) used to form an image from the secondary electrons emitted from the sample. A fluorescence detector (SiriusSD 265i C, RaySpec Ltd, UK) is located directly behind the SED to allow X-ray fluorescence to be detected from samples for anomalous scattering experiments. Precisely above the sample position, a PEEK (polyether ether ketone) guide tube is located, through which the gripper system can move vertically to mount or unmount a sample holder from the sample position.

The environment around the sample is extremely busy and allows multiple pieces of equipment to intersect or be directed at the sample position. The design of the sample vacuum vessel was carried out in-house, and was 3D-printed from aluminium alloy (AlSi_10_Mg, CA Models, UK) so that ports could be arranged to support and position all the required equipment. The exit window was sized to allow the wide-angle diffraction to exit the vessel (100° opening centred on the direct beam axis), and consists of a 125 µm Kapton film, positioned against an O-ring to form a seal. Just inside the vessel, but as close as possible to the Kapton window, sits a beamstop consisting of a carbon fibre arm with a 0.5 mm-diameter tungsten cup at one end to stop the direct X-ray beam. The carbon fibre arm is mounted onto two SmarAct compact piezo stages facilitating its alignment to the direct X-ray beam in the *x* and *y* planes.

### 2.4. Sample holders, sample hotel and gripper

**Figure 3:**
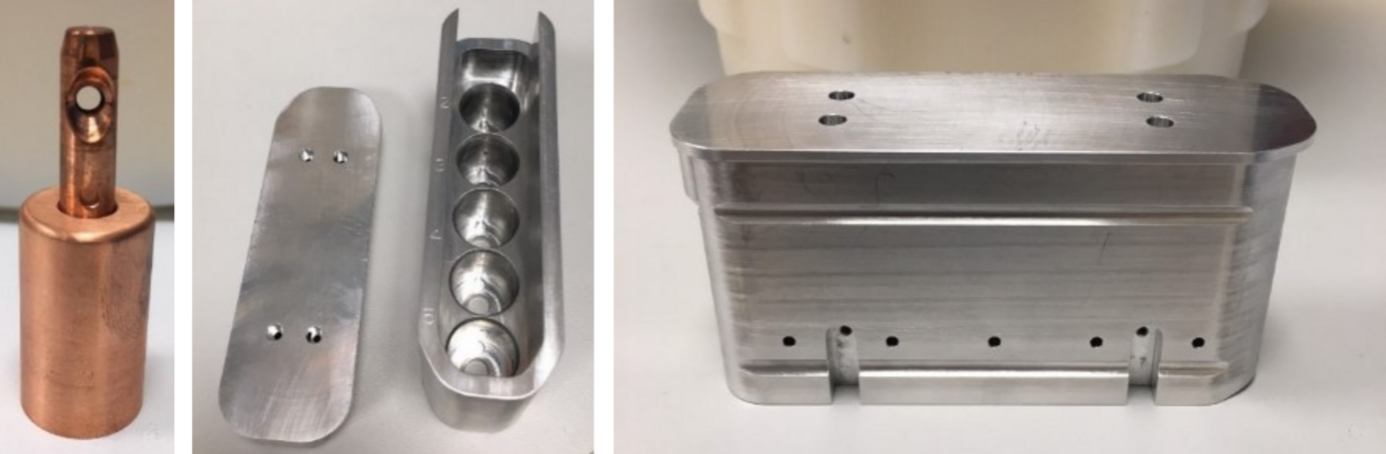
Picture showing the VMXm sample holder and sample cartridge. (*a*) An empty copper sample holder is shown, the empty opening holds a cryoEM grid, held in place by a c-clip. The opening to this is angled to allow diffracted X-rays to reach the detector and for as much rotation as possible for data collection. The base of the holder is a large copper mass to maintain the low temperature of the sample. (*b*) The open sample cartridge constructed from aluminium. This shows the five positions for holding each sample holder. (*c*) The closed sample cartridge. The cartridge has a lid to maintain low sample temperatures and reduce water ice formation on the samples while the cartridge is being transferred to the beamline. On the base of the cartridge two cutouts can be seen where the claw mechanism fits for transferring the cartridge from liquid nitro­gen into the airlock.

To avoid interference with the performance of the scanning electron microscope, it was essential to ensure that no magnetic parts were used in or around the sample environment. Furthermore, because the whole sample environment must operate under vacuum, a standard nitro­gen cryostream could not be adopted. These limitations ruled out using standard SPINE pins at the sample position. Instead, the cooling of the samples needed to be carried out via conductive cooling of the sample holders. To do this effectively, the sample holders were designed out of copper, due to its low specific heat capacity, and they rely on the large thermal mass of the holders to keep the sample temperature below the point of devitrification of water. The sample holders and sample cartridge are shown in Fig. 3 ▸. The sample holder is made from two parts of copper connected via a threaded screw, enabling interchangeability of different sample mounts. A 3 mm aperture is present in the holder where a standard cryoEM grid can be positioned; this is then held in place by a tungsten circlip (Agar Scientific, UK). It is possible to house up to five of these sample holders in an aluminium sample cartridge, which is then used to transfer the samples into the beamline endstation. Specifically, the sample holders are transferred to this cartridge under liquid nitro­gen in a specially designed foam Dewar containing a cutout to align the cartridge in this Dewar. The lid is then placed on top of the cartridge to help maintain the temperature of the samples below the glass transition temperature of water. The Dewar is taken to the beamline and placed in a Perspex chamber with flowing dry nitro­gen gas. From here, it is manually transferred out of the liquid nitro­gen, using a claw mechanism that grasps four cutouts at the base of the cartridge [Fig. 3 ▸(*c*)], to the airlock through the nitro­gen stream. The airlock can then be evacuated before opening a gate valve which allows the cartridge to be transferred into the VMXm endstation sample hotel. A video showing the loading procedure can be found in the supporting information. A magnet housed in the lid of the cartridge is attracted to a second magnet on the roof of the hotel as the cartridge is introduced, thus removing the lid from the cartridge. Once the cartridge is seated in the hotel, two liquid nitro­gen cooled copper plates are moved to clamp the cartridge on either side and maintain its temperature at cryogenic conditions. The cartridge can be translated horizontally to the required sample position where the gripper can move vertically into the top of the cartridge and grip the sample to be loaded. The gripper itself is actively cooled, which means the sample is also actively cooled while gripped. Once the required sample is gripped, the gripper moves up clear of the cartridge, to allow the cartridge to move inboard, exposing an opening below leading down to the sample position. The gripper is then safe to move down through this aperture and, when close to the sample position, the sample can be released and then gripped in place at the sample position. As the sample position is not currently actively cooled, the sample relies on the large copper mass of the holder to maintain its temperature below the glass transition temperature. This gives a window of approximately 5 min, in which the sample remains below 130 K, where data can be collected from crystals before the gripper has to be re-introduced to cool the sample again. During this time the sample drifts vertically up at a rate of 1.785 µm min^−1^. To compensate for this drift, a motor correction has been applied during data collection in which the sample is moved down at the same rate as this drift, keeping the sample aligned to the beam position during this time. Samples can be removed from the sample position and placed back in the cartridge using the reverse process. The sample gripper and hotel system are connected to the top port of the sample vessel and share a common vacuum.

### 2.5. Experiment control and data analysis

The low-level control of the VMXm beamline is achieved using the *Experimental Physics and Industrial Control System* (*EPICS*) framework (https://epics.anl.gov/). *EPICS* is a very well established and tested software framework which provides many modules to easily integrate different components into a unified control system.

For motion control, Diamond’s standard controller is the Geobrick LV (Faraday Motion Controls Ltd, UK), capable of controlling up to eight axes. VMXm utilizes 12 Geobricks dedicated to controlling the optics hutches and experimental hutch components.

The three basic types of motor used on VMXm are: stepper motors, servo motors and piezo motors. These are normally fitted with encoders to obtain accurate feedback information which can close the control loop. Two examples of piezo motors are in the microfocus mirrors and the sample stages. The microfocus mirrors are controlled with a step and direction output from a Geobrick to a PiezoMotor Microstep Driver 101 (PMD 101, PiezoMotor, Sweden). The sample stages are controlled in a similar way with the output from a Geobrick to a SmarAct SDC2 controller (SmarAct GmbH, Germany). Both piezo solutions allow fine positioning of the mirrors and sample stages, with a resolution of 1 nm for the encoders.

The GigE cameras used within the diagnostics and OAV system are supported in *EPICS* using the Aravis library (https://github.com/AravisProject/aravis) linked into the areaDetector *EPICS* support module.

For the EIGER and PILATUS detectors, these are interfaced to *EPICS* using the ODIN framework, which was developed at Diamond Light Source (Yendell *et al.*, 2019 ▸). The ODIN framework uses the open-source ZeroMQ messaging library (https://zeromq.org/) for fast data transfer.

To assist with the smooth running of the beamline for users, the *Generic Data Acquisition* (*GDA*, https://www.diamond.ac.uk/OpenGDA/) software is used as an interface to allow users to align crystals and collect data. This also includes the control of loading samples, and re-cooling during data collection. The user interface has been designed to be broadly similar to the other MX beamlines at Diamond for ease of use for the user. The main difference relates to the sample loading and cooling of samples.

After data are collected on the beamline, several auto-processing jobs are triggered on the Diamond cluster to give users a fast indication about their data processing statistics. These include *fastdp*, *xia2/3dii* (Winter, 2010 ▸; Kabsch, 2010 ▸), *xia2*/*dials* (Winter *et al.*, 2022 ▸) and *AutoProc* (Vonrhein *et al.*, 2011 ▸; Kabsch, 2010 ▸), with the results of these being displayed in *SynchWeb* (Fisher *et al.*, 2015 ▸), the ISPyB web interface (Delagenière *et al.*, 2011 ▸), when the processing is complete. Of great benefit to VMXm is the use of *xia2.multiplex* (Gildea *et al.*, 2022 ▸) which also runs automatically if data have been successfully processed using *dials*. It is then possible to monitor the merging of several data sets to observe when the required completeness and multiplicity are obtained, and to have live feedback about sample isomorphism and preferential orientation.

### 2.6. Beam stability and feedback

As samples and the beam cross section become smaller, the negative impact of beam instability increases. To reduce beam vibrations and intensity fluctuations to a minimum, a closed-loop feedback system locking the beam position at the secondary source has been implemented. This feedback loop measures the beam position just upstream of the secondary source and steers the monochromatic beam to maintain its position using the second hDCM crystal. This feedback is performed by a Beam Enhanced Stabilization Technology (BEST) system from CAENels, Italy. The beam position is measured by the XBPM1 adjacent to the secondary source slits and any corrections are applied by acting on the hDCM second crystal fine-pitch and fine-roll piezo actuators. Although the BEST system can run at up to 100 kHz, we have found the setup is most stable at 100 Hz, as we have found at frequencies above this vibrations from the HPFM influence the feedback loop.

**Figure 4:**
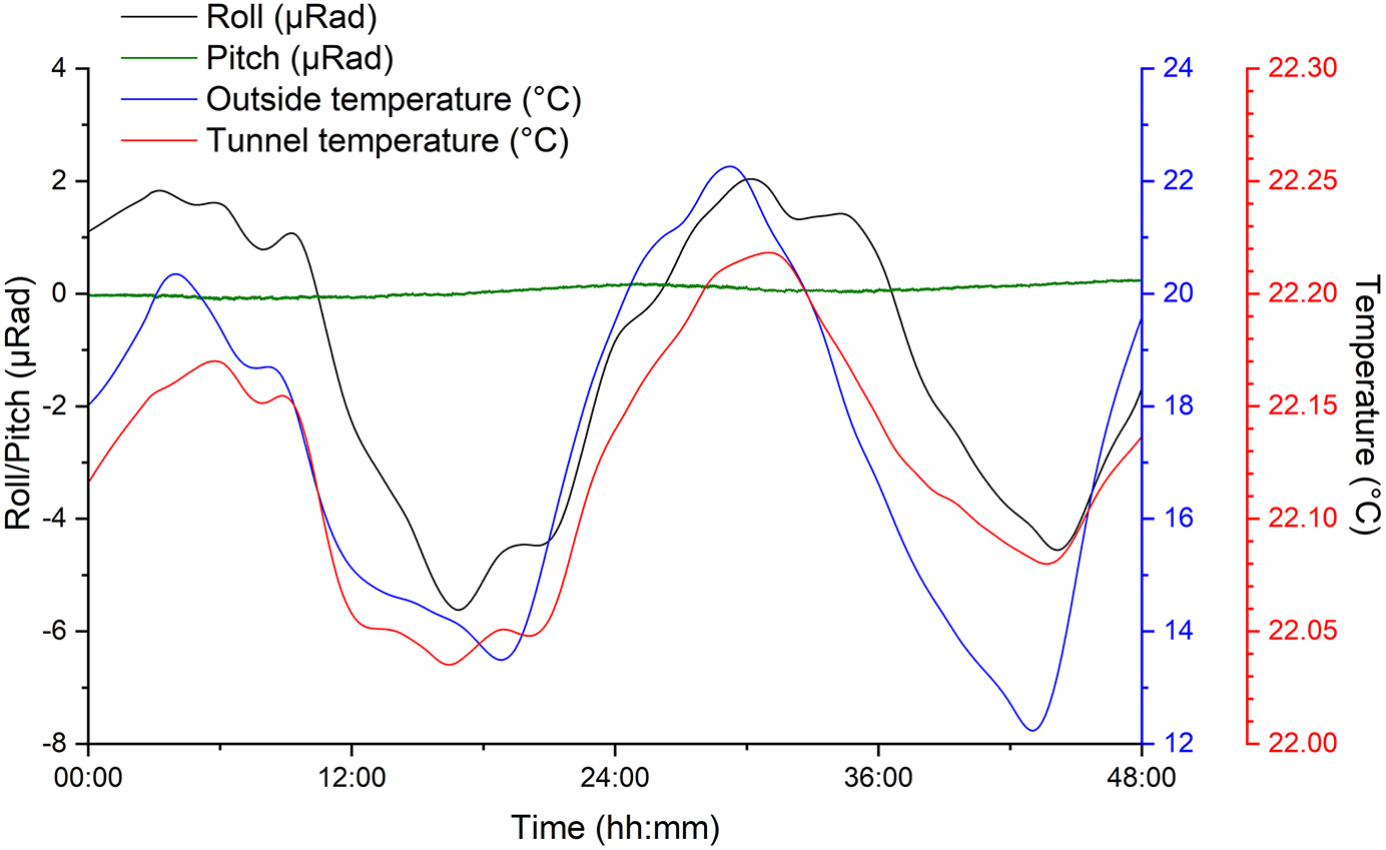
Plot of the movement in the hDCM pitch (green) and roll (blue) motors over the course of 48 h, and the corresponding outside air temperature (black) and temperature inside the storage-ring tunnel close to VMXm (red). The plots for roll and the temperatures have been smoothed for clearer analysis.

The movement of the fine-pitch and -roll piezos was monitored over a period of 48 h to understand the magnitude of the corrections on the hDCM while maintaining the beam centred on XBPM1. The movement of these was also compared with the outside air temperature, and the corresponding temperature in the storage-ring tunnel in the VMXm sector (Fig. 4 ▸). The change in roll (<7 µrad over 12 h) can be seen to correspond with the change in temperature, returning to approximately the same position after just over 24 h. The motion of pitch (∼0.2 µrad over 12 h) is of much smaller magnitude compared with roll.

Due to the large demagnification of the beam at the second focal stage, any vibrations will also be demagnified. To further improve beam stability, two XBPMs are positioned in the experimental hutch. The first is mounted before the fast shutter (XBPM2) and used to check beam position prior to the microfocusing mirrors, and the second just after the mirrors (XBPM3) very close to the sample position (60 mm). The beam is monitored on XBPM3 and any longer-term drifts are corrected for manually with moves in VMFM *Y* or HMFM *X*.

**Figure 5:**
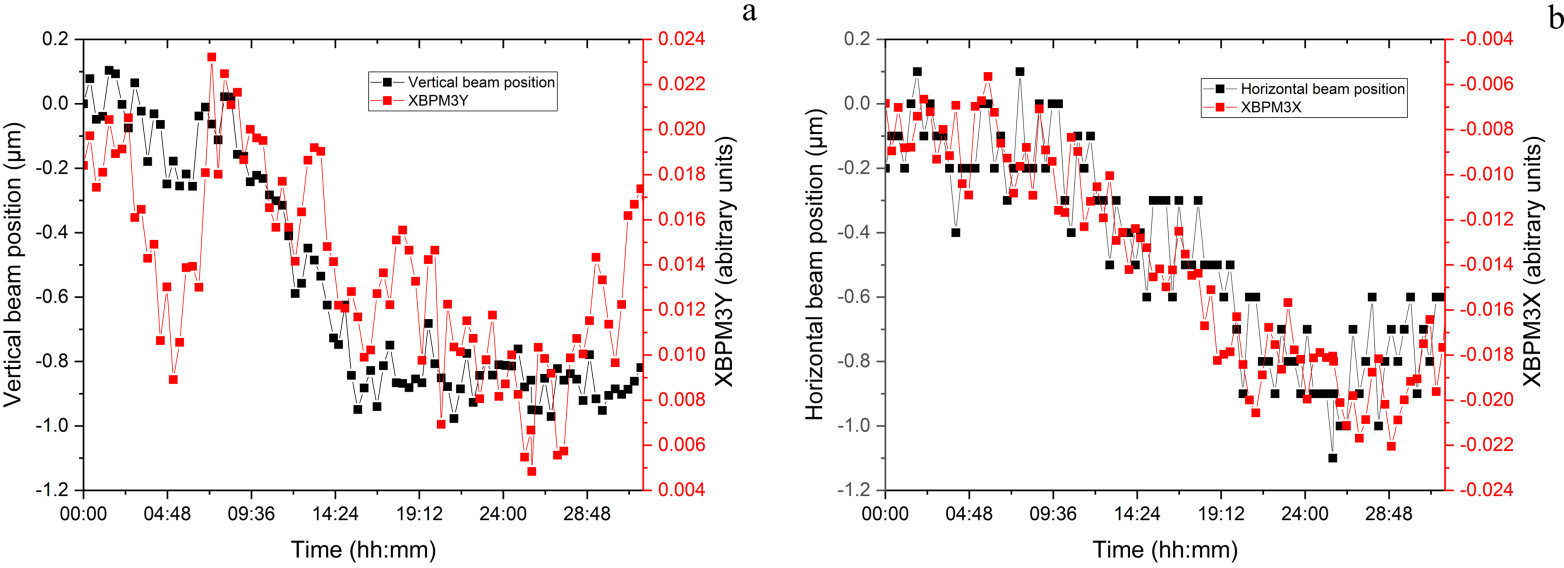
Beam position stability at the sample position, measured using knife-edge data (black) and monitoring of the position on XBMP3 (red) for the vertical (*a*) and horizontal (*b*). Measured over a 32 h period, with data points measured every 20 min.

These longer-term drifts have been monitored at the sample position, using repeated knife-edge scans in the horizontal and vertical axes measured at the sample position every 20 min for 32 h. For these measurements an energy of 21.299 keV was used, and a beamsize of 2.1 × 3.7 µm (v × h). The vertical and horizontal scans were measured using a gold knife-edge (Karlsruhe Institute of Technology, Germany) mounted at the sample position. This was scanned through the X-ray beam with the intensity measured on a silicon PIN diode (OSI Optoelectronics, USA) mounted just downstream of the sample position. The beam position was calculated by fitting a Gaussian peak to the derivative of the plot. The beam positions were then plotted against time and compared with the beam motion on XBPM3; the relevant plots can be seen in Fig. 5 ▸.

In both the horizontal and vertical the beam moves at the sample position by approximately 1 µm over the 32 h period, this is without any correction of the beam position using the HMFM and VMFM. Over a shift (8 h), in which data collections would take place, the beam motion is less than 1 µm. This motion at the sample position correlates well with the beam motion observed on XBPM3. With manual corrections of the VMFM and HMFM to maintain beam position centred on XBPM3, the beam position can be maintained at the sample position, allowing for data collections to date on crystals down to ∼1 µm in size.

## 3. Sample preparation and characterization facilities

VMXm sample holders have been designed to accept standard 3 mm-diameter cryoEM grids and maintain them at temperatures below the glass transition temperature of water. Notably, the current sample holder design limits the rotation range of crystals in the beam to approximately 90° at the centre of the grid. Higher rotation angles result in masking of the diffracted X-rays by the sample mount.

The VMXm sample preparation laboratory is fully equipped with a glow discharging device (EasiGlow, Pelco, USA), plunge freezer (EM GP2, Leica, Germany), scanning electron microscope (JSM-IT100, Jeol, Japan) fitted with a cryo-transfer/stage system (PP3006, Quorum, UK) and bespoke tools to aid loading of grids into the VMXm endstation.

CryoEM grids can be prepared in the VMXm sample preparation laboratory, or at home laboratories if similar equipment is available. At VMXm, slurries of crystals are mounted on copper cryoEM grids with holey carbon support films from Quantifoil, Germany, before single-sided blotting and flash-cooling in liquid ethane using a Leica EM GP2 plunge freezer (Crawshaw *et al.*, 2021 ▸). Grids are then stored under liquid nitro­gen using cryoEM grid storage boxes and pucks. The quality of the grids can be assessed prior to beamtime using the VMXm laboratory-based scanning electron microscope fitted with a cryo-stage.

## 4. Results

### 4.1. Beamsize

Measurements of the beamsize were carried out at 12.458 keV; however, it is similar over the entire energy range.

**Figure 6:**
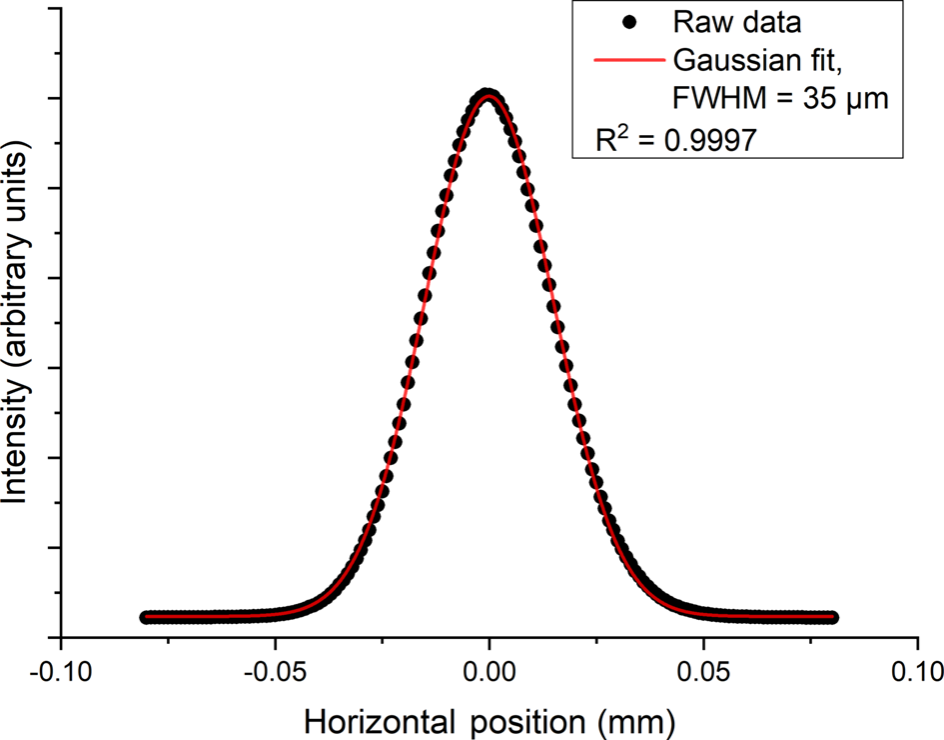
Secondary source horizontal beam profile. Raw data are shown as filled circles, with the fitted Gaussian shown as a line plot. The beamsize from the fit was calculated as 35 µm (FWHM).

Pencil beam scans were carried out on the HPFM by using a 0.01 mm horizontal slit just upstream of it. The beam profile was imaged on the scintillator directly after the secondary source, with the S3 slits fully open. The scintillator is positioned 238 mm downstream of the secondary source slits. The collected measurements were then used to calculate which voltages were to be applied to the HPFM to deliver the optimum focus at the secondary source slits. Fig. 6 ▸ shows the horizontal beam profile at the secondary source collected via a slit scan of the horizontal slit, with the intensity being measured on a silicon PIN diode (OSI Optoelectronics, USA). A Gaussian was fitted to the profile, giving a horizontal beamsize of 35 µm, as measured by the full width at half-maximum (FWHM) of the peak.

The vertical and horizontal beamsizes at the sample position were measured using gold knife-edge scans, similar to those used to monitor the beam stability. Here the calculated beamsize (FWHM) was determined by fitting a Gaussian peak onto the derivative of the plot.

**Figure 7:**
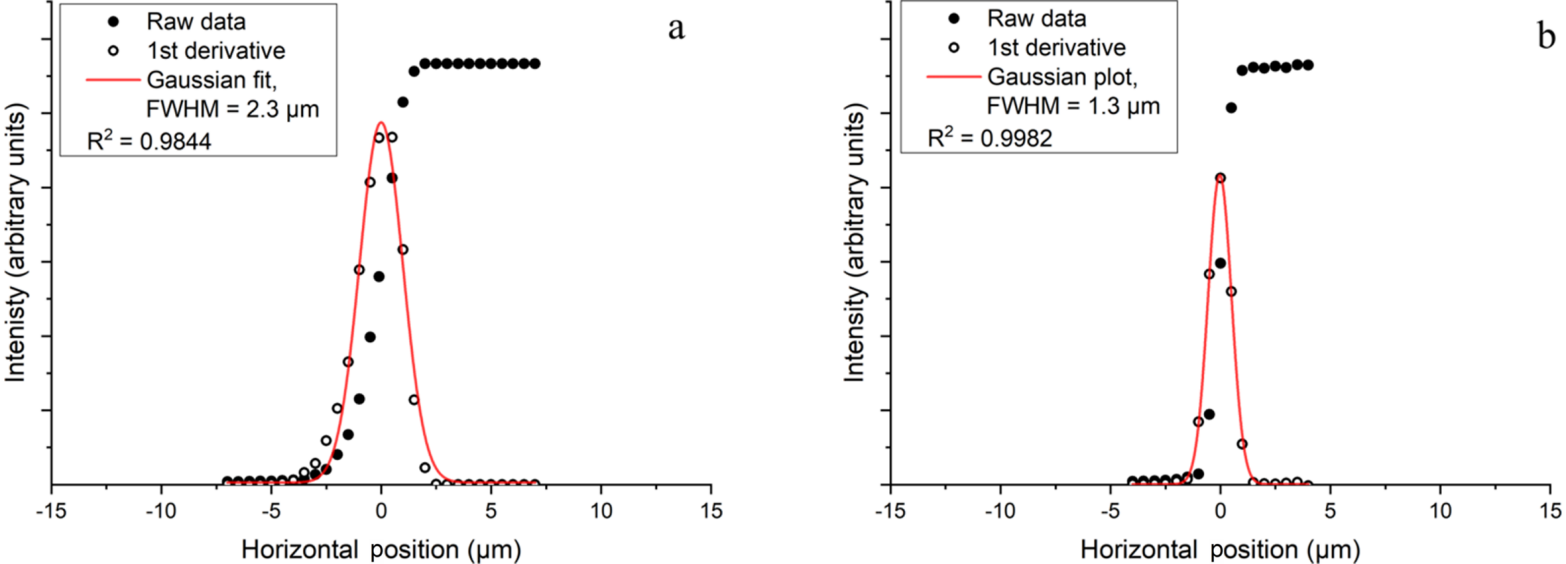
Horizontal beam profile at the sample position. The raw data from the knife-edge scan are displayed as filled circles, the first derivative of the knife edge is shown as open circles and the Gaussian fit to this derivative is shown as a line plot. (*a*) Data collected with the secondary source slits fully open at 0.07 mm show the sample position has a measured beamsize of 2.3 µm (FWHM). (*b*) Data collected with the secondary source slits closed to 0.005 mm show the measured beamsize at the sample position reduces to 1.3 µm (FWHM).

For the horizontal scans, the knife-edge was scanned horizontally through the beam, with the intensity being recorded on a diode. For transmission of the full beam, the secondary source slits were set to 0.07 mm. This setting trimmed the tails of the 35 µm beam at the secondary source. The horizontal beamsize achieved at the sample position was 2.3 µm (FWHM). Closing the secondary source slits to 0.005 mm reduced the beamsize to 1.3 µm (FWHM) (Fig. 7 ▸).

**Figure 8:**
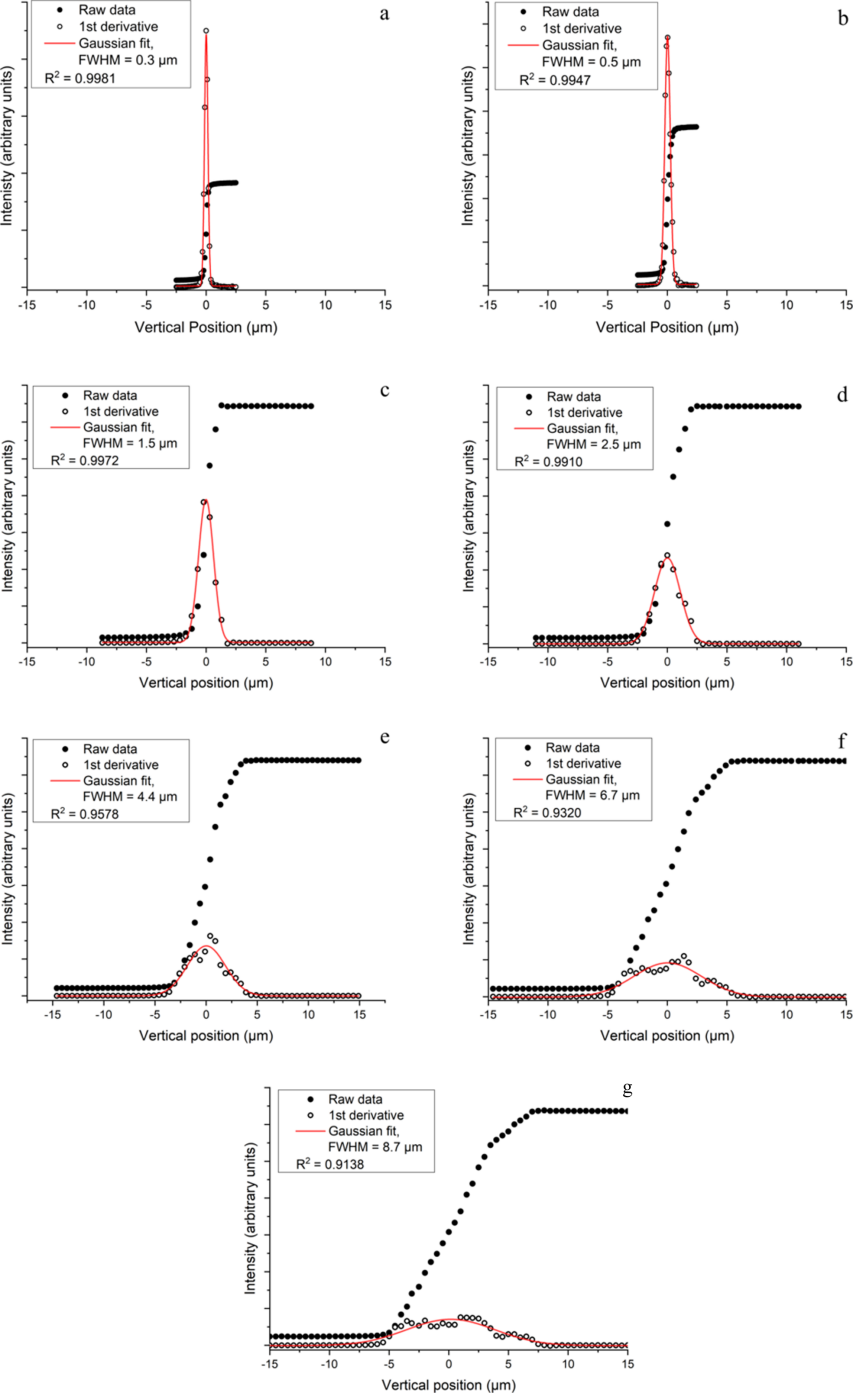
Vertical beam profile for the seven lanes of the VMFM at the sample position. The raw data from the knife-edge scan are displayed as filled circles, the first derivative of the knife-edge is shown as open circles and the Gaussian fit to this derivative is shown as a line plot. All plots show the FWHM of the measured beamsize, compared with the calculated beamsizes for (*a*) lane one, 0.3 µm, (*b*) lane two, 0.5 µm, (*c*) lane three, 1.5 µm, (*d*) lane four, 2.5 µm, (*e*) lane five, 4.4 µm, (*f*) lane six, 6.7 µm, (*g*) lane seven, 8.7 µm.

To measure the vertical beamsize at the sample position, the knife-edge was scanned vertically through the beam using either the coarse (stepper motor) or fine (piezo stack) vertical motors, depending on the lane of the VMFM and size of the corresponding beam and step size required. The derivative was taken of the raw knife-edge data, and a Gaussian peak was fitted to these data to give a FWHM measurement of the beam profile (Fig. 8 ▸). The vertical beamsizes measured and fitted for each of the lanes were 0.3, 0.5, 1.5, 2.5, 4.4, 6.7 and 8.7 µm (FWHM). The overlay of the first derivative of each of the seven lanes, illustrating the comparative changes in beamsize and intensity, are shown in Fig. S1 of the supporting information.

### 4.2. Background

#### 4.2.1. Differences in background compared with a standard MX beamline

To investigate the effect of vacuum on background photon counts, diffraction data of crystals of cypovirus *Lymantria dispar* 14 (CPV14) polyhedra (2–4 µm in size) were compared when collected on beamline I24 (in air) at Diamond Light Source and at VMXm (in vacuum). Crystals of CPV14 were mounted on cryoEM grids, blotted and flash-cooled in the same manner for both beamline experiments. On VMXm the grids were held under vacuum and conductively cooled, while on I24 they were held in air, cooled with a nitro­gen cryostream. The diffraction-weighted dose (Zeldin, Brockhauser *et al.*, 2013 ▸) in *RADDOSE-3D* (Zeldin, Gerstel *et al.*, 2013 ▸) was used as a measure to calculate the radiation dose for both VMXm and I24 to ensure that this was equivalent during data collection at 21.326 keV. The incident flux and energy of the two beamlines were taken into consideration, and exposure times altered accordingly to give the equivalent dose per exposure. For all experiments 100% transmission of the beam was used, with 0.1° rotation width per frame and a detector distance of 313 mm. For VMXm a beamsize of 3 × 3 µm was used with an exposure time of 0.1 s per image for both energies. For I24 the beamsize was 10 × 10 µm, and the exposure time changed to ensure both experiments received the equivalent dose. For the data collection at 21.326 keV the exposure was 0.125 s (19 MGy per image).

**Figure 9:**
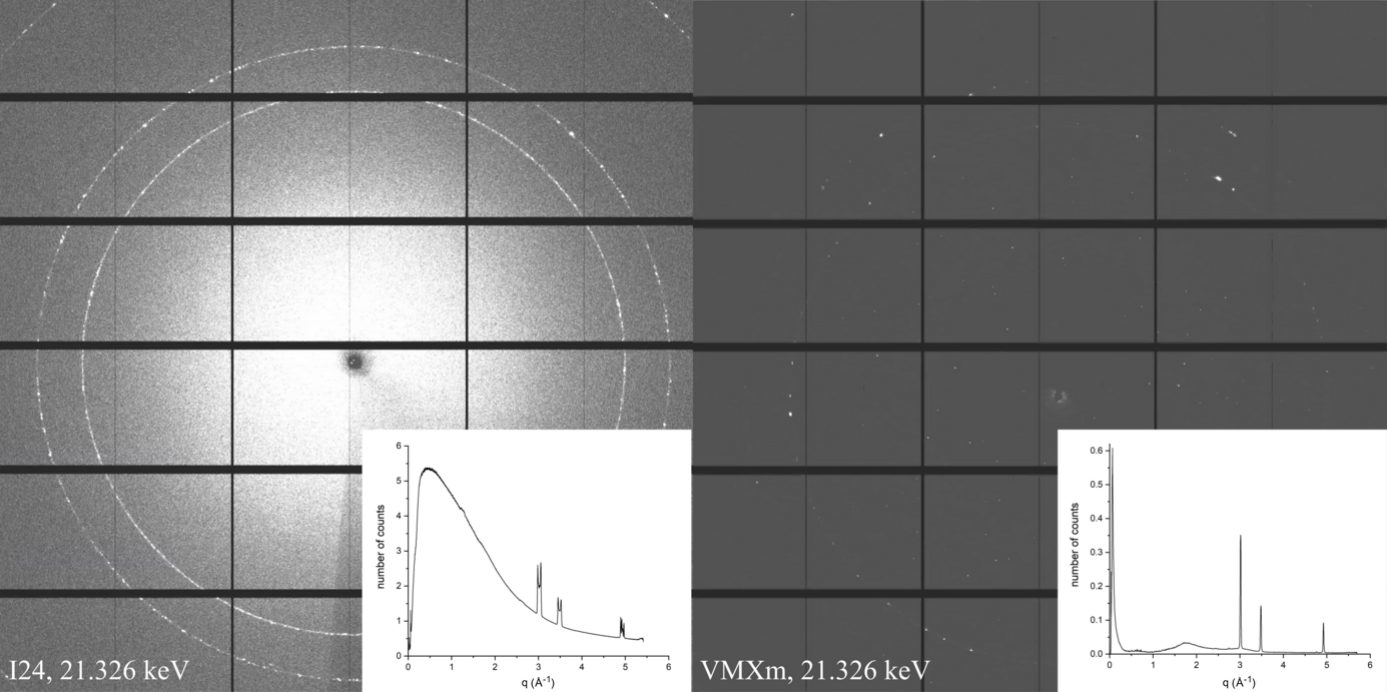
Comparison diffraction images of data collected on CPV14 on I24 and VMXm at 21.326 keV. Diffraction images have been visualized using *dui2* and scaled similarly to give equivalent views of each image. An azimuthal integration was taken through each diffraction data set to give an equivalent average number of photon counts for each series of measurements; this can be seen as the inset panel in each image. The peaks in the traces around 3, 3.5 and 5 Å^−1^ are copper powder diffraction rings from the copper cryoEM grids.

The difference in the background between the two beamlines is shown in Fig. 9 ▸. In this figure, representative diffraction images are shown for both I24 and VMXm, plotted using *dui2* (Fuentes-Montero *et al.*, 2016 ▸), with images scaled similarly to give equivalent views of the image. To understand the difference in the number of counts between these data sets, *DAWN* (Filik *et al.*, 2017 ▸) was used to take an azimuthal integration of the data, giving an average number of counts per image versus *q* (Å^−1^). Plots of these data are shown inset to each diffraction image in Fig. 9 ▸. In general, the in-air measurement carried out on I24 shows much higher background photon counts closer to the beamstop which tail off at higher scattering angles (∼6 counts). In contrast, for the equivalent measurement in vacuum on VMXm, the average number of background photon counts remains below 0.1. The exception to this is the low-*q* peak visible up to ∼0.6 counts due to beam scatter around the beamstop, possibly indicating that the beamstop needs to be larger to capture this.

#### 4.2.2. VMXm X-ray background measurements

To quantify the impact of sample environment and energy on total measured X-ray background on VMXm, several data sets were collected under varying conditions. To assess the difference in background for data collected in air compared with under vacuum, two data sets were collected at 21.326 keV (1.6 × 10^11^ photons s^−1^), with no sample mounted, one at ambient pressure and one in vacuum (∼5 × 10^−4^ mbar). For these data collections, a total of 1000 images were collected on the EIGER2 X 9M CdTe detector with an exposure time of 0.2 s, 100% transmission of the beam, 0.01° frame width and with the detector distance at 313 mm. To assess the effects of energy on the observed X-ray background, measurements on CPV14 crystals from Section 4.2.1 were used. Here measurements were collected on the EIGER2 X 9M CdTe detector at 12.458 keV (1.3 × 10^12^ photons s^−1^) and 21.326 keV (1.6 × 10^11^ photons s^−1^). For the 12.458 keV data set, a total of 100 images were collected with an exposure time of 0.1 s, 100% transmission of the beam, 0.1° frame width and with the detector distance set at 313 mm. For the 21.326 keV data set, all the parameters were the same, but a total of 500 images were collected. To analyse the data, the open-source software package *DAWN* was used (Filik *et al.*, 2017 ▸). In *DAWN* the processing pipeline was utilized to first mask out the module gaps from the EIGER and the beamstop. The images were then averaged, and an azimuthal integration was taken, plotting the number of counts per second per image versus *q* (Å^−1^).

**Figure 10:**
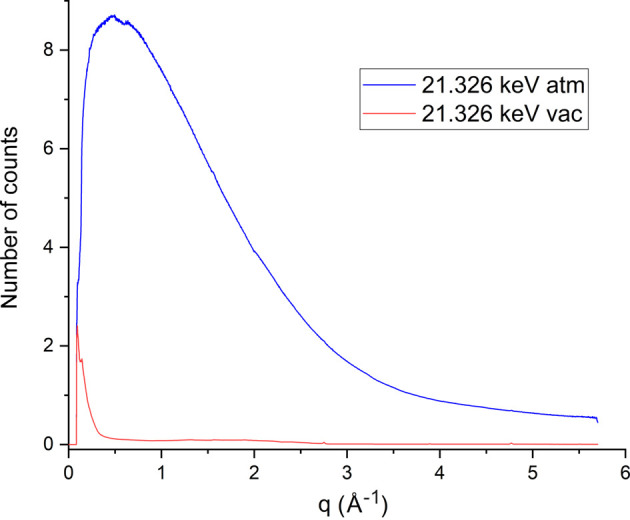
Graph displaying the average number of photon counts per second on an EIGER2 X 9M CdTe detector for a given resolution (*q*) at 21.326 keV on VMXm when at atmospheric pressure (blue trace) or under vacuum (red trace).

Fig. 10 ▸ shows a plot of the difference in the number of background counts per second at 21.326 keV when at atmospheric pressure or under vacuum. For these measurements the reduction in average background counts per second when the sample environment is placed under vacuum is instantly apparent. Almost zero counts are recorded over the entire *q* range, except for a small low-*q* peak close to the beamstop. This is compared with the measurement in air where there is a rise in low-*q* counts, up to around 9 counts per second, which then slowly drops off as the scattering angle increases. Even when the background plateaus at atmospheric pressure, this is approximately 0.5 counts per second higher than with a similar scattering angle when under vacuum.

**Figure 11:**
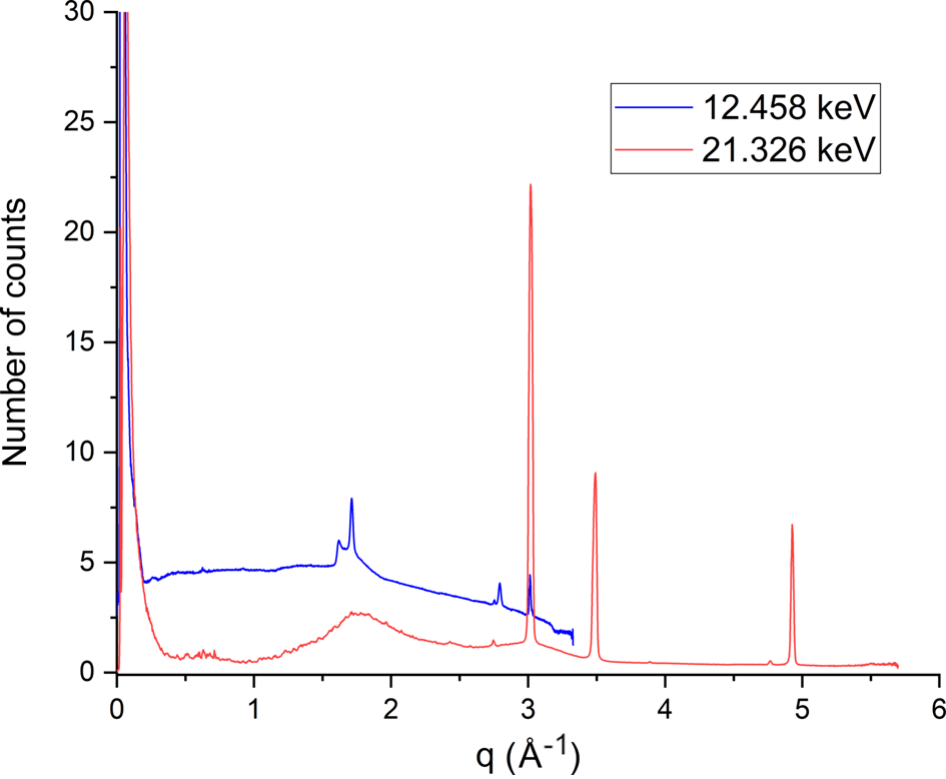
Plot comparing the difference in the number of photon counts per second on an EIGER2 X 9M CdTe at VMXm for crystals of CPV14 under vacuum at 12.458 keV (blue trace) and 21.326 keV (red trace). The peaks in the traces around 3, 3.5 and 5 Å^−1^ are copper powder diffraction rings from the copper cryoEM grids. Ice rings are also observed at a resolution of 1.7 and 2.7 Å^−1^ in the 12.458 keV, with these appearing as more diffuse rings in the 21.326 keV plot.

The difference in the number of photon counts per second for crystals of CPV14 under vacuum at 12.458 keV (blue trace) and 21.326 keV (red trace) can be seen in Fig. 11 ▸. Each energy produced a different flux, so all plots were normalized to the flux at 12.458 keV. The number of photon counts per second for 21.326 keV was below 0.5, except at low *q*, close to the scatter around the beamstop, and also around 1.7 Å^−1^ where a diffuse solvent/ice ring is observed. In comparison with the plot at 12.458 keV, here the average photon counts per second are around 4, with more defined peaks at a resolution of 1.7 and 2.7 Å^−1^, attributed to crystalline ice rings. Again, like at 21.326 keV, there is a larger peak in photon counts at low *q* due to the scatter around the beamstop. As the energy decreases, the X-ray absorption cross section increases, and hence an increase in the number of background photon counts was also observed. This cements the use of higher energies, not only to aid with photoelectron escape to improve crystal lifetime, but also leading to a further reduction in background photon counts.

### 4.3. Samples

#### 4.3.1. Comparison data collection on VMXm

**Figure 12:**
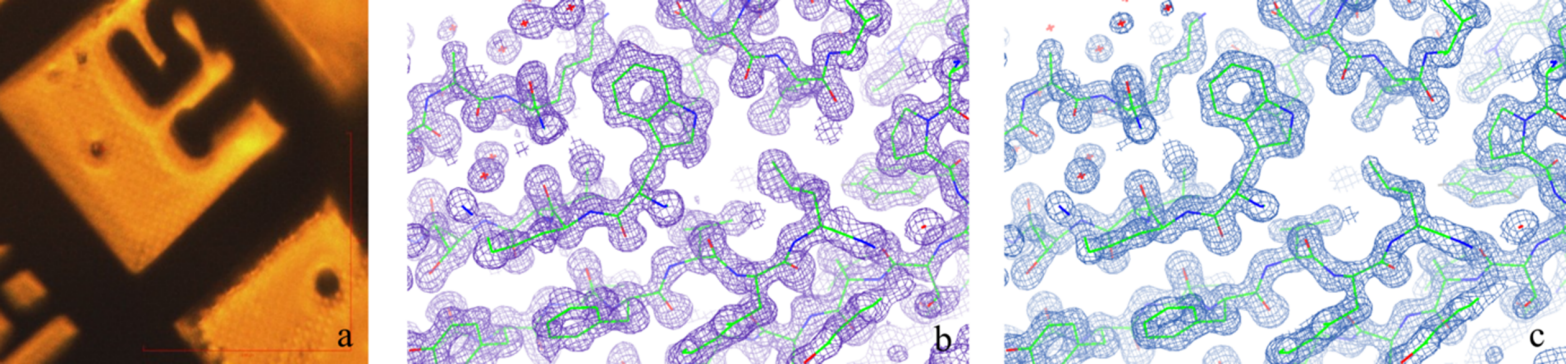
Data collection and structure solution of CPV14 polyhedra crystals on VMXm. (*a*) Crystals of CPV14 mounted on a cryoEM grid and displayed on the VMXm on-axis viewing system. The spacing between grid bars is approximately 100 µm, and the crystals range in size from 2 to 4 µm. (*b*) The *F*_o_ − *F*_c_ map contoured at 1.5σ for the structure of a single crystal of CPV14 on VMXm and (*c*) the *F*_o_ − *F*_c_ map contoured at 1.5σ from a merged data set from 14 crystals of CPV14 on VMXm.

Crystals of CPV14 were prepared as above (Fig. 12 ▸) and collected on VMXm for comparison with previously published data collected on I24 (Ji *et al.*, 2015 ▸). For the previously collected I24 data, polyhedra were applied to a MicroMesh mount and flash-cooled in a nitro­gen gas stream at 100 K. The data were collected at an energy of 12.664 keV and beamsize of 6 × 6 µm (using beam-defining slits) on a PILATUS 6M Si detector. Data were collected from 20 crystals and merged to obtain a complete data set. The data for VMXm were collected at 21.326 keV using the EIGER2 X 9M CdTe detector and a fully focused beam of 3.6 × 3.6 µm at temperatures below 130 K.

As the original raw I24 data were unavailable, we relied on the published statistics for comparison [*I*/σ(*I*) cut-off of 1.8 in the highest-resolution shell]. Therefore, a more recent data collection was performed on I24 using the same batch of crystals as those used for the VMXm study, mounted on a MicroMesh mount similar to the Ji *et al.* paper. The data here were collected at 12.800 keV on a PILATUS3 6M Si detector with a 6 × 6 µm beam (beam-defining slits) at 100 K. A total of 39 data sets were collected, with all wedges of data merged together using the same processing protocol as the VMXm data, with a CC_1/2_ cut-off of 0.3.

Using diffraction data collected on VMXm from a single polyhedra crystal, *xia2/dials* (Winter *et al.*, 2022 ▸) was used to process and scale the data, and the structure of CPV14 was solved by molecular replacement using the structure of CPV19 [Protein Data Bank (PDB) code 5a99] as a search model using *Phaser* (McCoy *et al.*, 2007 ▸). Although the resulting solution had *R*/*R*_free_ values of 0.53/0.56, these improved rapidly with *Buccaneer* (Cowtan, 2006 ▸), indicating a correct solution. Some regions of the density appear noisy [Fig. 12 ▸(*b*)], and the model refinement using *Phenix* (Liebschner *et al.*, 2019 ▸) does not improve beyond 0.229/0.257 *R*/*R*_free_. This is likely due to the lack of completeness (overall 90.69%, highest-resolution shell 57.69%, see Table 4 ▸) and radiation damage [as observed through plots of *R*_cp_ versus dose from the *dials* output (Winter *et al.*, 2019 ▸)], a calculated total dose of 24 MGy (calculated using *RADDOSE-3D*). The structure from the single-crystal data has been deposited in the PDB with the code 8qqc.

A more complete data set (overall 96.6%, highest-resolution shell 98.6%) was obtained by merging several wedges of data collected from 14 crystals (from a total of 25 data wedges collected) using *xia2.multiplex* (Gildea *et al.*, 2022 ▸). Exploiting the high multiplicity of these data, this data set could be filtered in *xia2.multiplex* using CC_1/2_ filtering with a stringent cut-off (deltacchalf.stdcutoff = 0.5 instead of the default 3) and using groups of ten images in order to minimize the effects of radiation damage. The structure of CPV14 was again solved by molecular replacement using the structure of CPV19 (PDB code 5a99) as a search model and *Phaser*. The model of the refined data gave values of 0.15/0.18 for *R*/*R*_free_. The PDB deposition for this structure is 8qph. The electron-density maps for both the single-crystal data, and the 14 merged data sets can be seen in Fig. 12 ▸. Although very similar, the multi-crystal structure yielded an overall map–model correlation of 0.942 while that for the single-crystal data was only 0.907 overall (as reported by *Phenix*).

A comparison of the statistics between the two VMXm data sets and the data sets collected on I24 is provided in Table 4 ▸. Re-processing of the newer I24 data, using *xia2/dials* with a CC_1/2_ cut-off of 0.3, improved the resolution from 1.91 Å to 1.64 Å. The data statistics for VMXm demonstrate that, through multiple improvements in sample preparation and data collection, significant structural information could be obtained from a single 3–4 µm crystal. The crystals were mounted more optimally, minimizing the liquid surrounding them, which led to lower background from the surrounding mother liquor. The fully focused beam cross section was better matched to the crystal size, further improving signal-to-noise. Based on previous work (Storm *et al.*, 2020 ▸; Storm *et al.*, 2021 ▸), it is also assumed that data collected at higher energies exploited photoelectron escape from the 3–4 µm-sized crystals. This was further optimized by using the EIGER detector with a CdTe sensor, which has higher quantum efficiency at these energies, ensuring that as much signal as possible was collected from the weak diffraction. With these factors in mind, not only was a data set with an overall completeness of 90.5% collected from a single crystal, but when merging data from multiple crystals, the resolution limit was also improved from 1.64 Å on I24 to 1.30 Å on VMXm in this study.

## 5. Facility access

The VMXm beamline is accessible through the standard Diamond peer-reviewed proposal system. It is recommended that sample suitability and preparation are discussed with beamline staff prior to proposal submission so that assistance and support can be offered. Beamtime can also be obtained on a commercial basis by contacting the Diamond Industrial Liaison Office.

## 6. Discussion and conclusions

The newest addition to the suite of MX beamlines at Diamond Light Source is VMXm. We have presented here how the beamline has been designed and constructed to allow rotation data to be collected from microcrystals less than 10 µm in size.

The beamline to date has been measuring diffraction data on crystals down to ∼1 µm in size. This has been made possible through the design and stability of the beamline. Many of the crystal vibrations, and hence sources of instrument error (Alkire *et al.*, 2008 ▸; Flot *et al.*, 2006 ▸), which would normally be encountered due to the presence of a cryostream, have been eliminated through the use of the *in vacuo* sample environment. The samples are currently cooled via conductive cooling by the gripper. This gives a ∼5 min window for data collection before the gripper needs to re-cool the sample. A current project is in place to upgrade the goniometer to also be actively cooled, which should then open many more opportunities for VMXm.

The optics and endstation setup allow microcrystals to be probed with the full beam as small as 0.3 × 2.3 µm (vertical × horizontal), housing the sample in vacuum to limit any background scatter. These properties, along with the careful mounting of crystals on cryoEM grids with minimal liquid coupled with using higher X-ray energies, typically allow optimum low-background diffraction data to be collected on microcrystals. Furthermore, the use of the EIGER CdTe detector, and data collection at higher energies presumably also increases the lifetime of the crystals based on previous work (Storm *et al.*, 2020 ▸; Storm *et al.*, 2021 ▸). These gains allow for the resolution limit of data collections to be improved, and for more data to be collected from each single crystal. The beamline enables the most challenging of microcrystals to be studied, which would not have been possible previously.

## Supplementary Material

Video of sample loading. DOI: 10.1107/S1600577524009160/rv5180sup1.mov

Figure S1. DOI: 10.1107/S1600577524009160/rv5180sup2.pdf

PDB reference: Crystal structure of Lymantria dispar CPV14 polyhedra single crystal, 8qqc

PDB reference: Crystal structure of Lymantria dispar CPV14 polyhedra 14 crystals, 8qph

## Figures and Tables

**Table 1:** Diamond Light Source double-double-bend achromat (DDBA) source, beamline VMXm X-ray beam, insertion device and front-end parameters

Ring current	300 mA
Photon source	2 m-long 21 mm-period undulator
Photon source parameters @ 12.4 keV	σ′_h_ = 42 µrad (FWHM = 98 µrad)
	σ′_v_ = 9 µrad (FWHM = 21 µrad)
	σ_h_ = 84 µm (FWHM = 197 µrad)
	σ_v_ = 4.3 µm (FWHM = 10.1 µrad)
Front-end aperture acceptance	109 × 37 µrad

**Table 2:** VMXm beamline components and their distance from the source

Beamline component	Acronym	Distance from source (m)
White beam slits	S1	25.00
White beam diagnostics	D1	26.65
Horizontal deflecting double crystal monochromator	hDCM	29.85
Diagnostic	D2	31.52
Horizontal slits	S2	31.92
Horizontally deflecting mirror	HPFM	32.50
X-ray beam position monitor	XBPM1	38.31
Secondary source slits	S3	38.50
Diagnostic	D3	38.71
Attenuators	Att	47.60
X-ray beam position monitor	XBPM2	48.30
Fast shutter	FS	48.34
Slits	S4	48.70
Horizontally deflecting mirror	HMFM	49.20
Vertically deflecting mirror	VMFM	49.60
X-ray beam position monitor	XBPM3	49.94
Slits	S5	49.95
Slow shutter	SS	49.95
On-axis viewing system	OAV	49.98
Sample	Samp	50.00

**Table 3:** Beamline VMXm optical parameters RMS denotes root mean square.

	HPFM	HMFM	VMFM (lane 1)
Distance from source (m)	32.5	49.2	49.6
Reflection direction	Inboard	Outboard	Up
Shape	Tangential ellipse; 16 element adaptive bimorph	Tangential ellipse	Tangential ellipse
*P* object distance (m)	32.5	10.7	49.6
*Q* image distance (m)	6.0	0.8	0.4
Optic active area (L × W, mm)	380 × 35	420 × 10	(310 × 4) × 7 lanes
Angle of incidence (mrad)	3.0	3.0	3.0
Coating	Rh, Pt and Si lanes	Rh (5 nm) over Pt (50 nm)	Rh (5.6 nm) over Pt (55 nm)
Substrate	Silicon	Silicon	Silicon
Tangential slope error (µrad RMS)	0.307 (max. correction ±150 V)	0.054	0.080
Roughness (nm RMS)	0.141	0.100	0.128

**Table 4:** Comparison of data processing statistics from CPV14 data collected on I24 from Ji *et al.* (2015 ▸) merging 20 crystals and on MicroMeshes merging 39 crystals, and on VMXm with data from one crystal and with merging from 14 crystals The values in parentheses relate to the highest-resolution shells for each data set.

	Ji *et al.*, I24	I24 – MicroMesh	VMXm – 1 crystal	VMXm – 14 crystals
Detector	PILATUS 6M Si	PILATUS3 6M Si	EIGER2 X 9M CdTe	EIGER2 X 9M CdTe
No. crystals	20	39	1	14
Energy (keV)	12.664	12.800	21.326	21.326
Beamsize (µm)	6 × 6 (with slits)	6 × 6 (with slits)	3.6 × 3.6	3.6 × 3.6
Resolution (Å)	72.5–1.91 (2.02–1.91)	72.83–1.64 (1.67–1.64)	42.1–1.30 (1.32–1.30)	72.97–1.34 (1.39–1.34)
Unique reflections	12952 (1045)	21282 (1111)	40615 (1275)	39736 (4090)
Redundancy	7.9 (1.6)	29.0 (9.4)	4.5 (2.0)	43.3 (15.6)
Completeness (%)	92.3 (52.1)	94.9 (100)	90.7 (57.7)	96.6 (98.6)
*I*/σ(*I*)	8.4 (1.8)	7.9 (1.0)	3.70 (0.54)	9.4 (0.9)
Wilson *B* factor (Å^2^)	10.86	9.48	16.59	8.36
*R*_merge_	0.199 (0.327)	0.437 (2.783)	0.304 (0.910)	1.369 (2.919)
CC_1/2_	N/A	0.991 (0.279)	0.911 (0.356)	0.867 (0.302)
*R*_work_	0.133 (0.136)	0.133 (0.221)	0.229 (0.347)	0.156 (0.355)
*R*_free_	0.194 (0.206)	0.161 (0.274)	0.257 (0.348)	0.180 (0.382)
Average *B* factor (Å^2^)	13.0	15.2	10.4	18.4

*R*_merge_ = 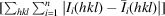/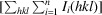, *R* = 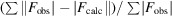; *R*_free_ was calculated using 10% of data excluded from refinement.
