# Supplementary material for: VMXm – A sub-micron focus macromolecular crystallography beamline at Diamond Light Source
Source: J Synchrotron Radiat. 2024 Oct 30;31(Pt 6):1593–608. doi: 10.1107/S1600577524009160 (PMC11542661; doi:10.1107/S1600577524009160)
Supplement: Supplementary file 2 [file s-31-01593-sup2.pdf]

JOURNAL OF SYNCHROTRON RADIATION

Volume 31 (2024)

Supporting information for article:

VMXm – A sub-micron focus macromolecular crystallography beamline at Diamond Light Source

Anna J. Warren, Jose Trincao, Adam D. Crawshaw, Emma V. Beale, Graham Duller, Andrew Stallwood, Mark Lunnon, Richard Littlewood, Adam Prescott, Andrew Foster, Neil Smith, Guenther Rehm, Sandira Gayadeen, Christopher Bloomer, Lucia Alianelli, David Laundy, John Sutter, Leo Cahill and Gwyndaf Evans

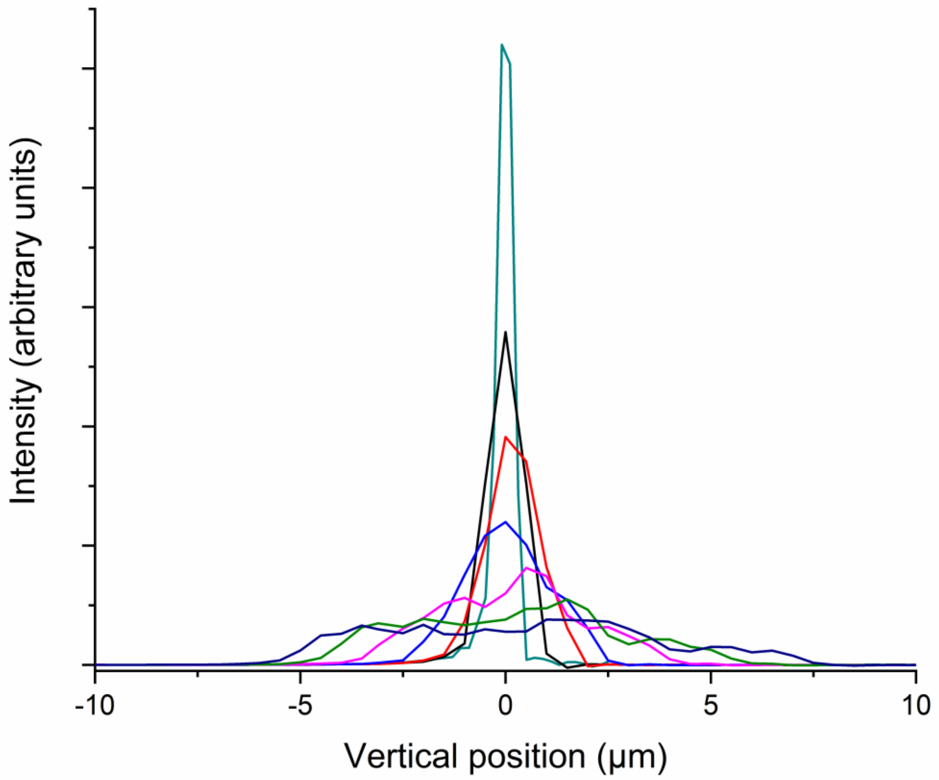

Figure S1 Overlay of the 1st derivative of data collected from knife-edge scans for each of the 7 lanes of the VMFM. The beamsize of these lanes when fitted with a Gaussian range from 0.3 µm (dark cyan), 0.5 µm (black), 1.5 µm (red), 2.5 µm (blue), 4.4 µm (magenta), 6.7 µm (green) to 8.7 µm (navy).
